# Absolute PET Myocardial Blood Flow Quantification in the Diagnosis and Risk Stratification of Coronary Microvascular Dysfunction

**DOI:** 10.31083/RCM53499

**Published:** 2026-10-12

**Authors:** Runze Wen, Dan Li, Xuemei Wang

**Affiliations:** ^1^Department of Nuclear Medicine, The First Affiliated Hospital of USTC, Division of Life Sciences and Medicine, University of Science and Technology of China, 230001 Hefei, Anhui, China; ^2^Department of Cardiovascular Medicine, The First Affiliated Hospital of USTC, Division of Life Sciences and Medicine, University of Science and Technology of China, 230001 Hefei, Anhui, China

**Keywords:** coronary microvascular dysfunction, myocardial flow reserve, positron emission tomography, myocardial blood flow

## Abstract

Invasive coronary angiography reveals non-obstructive coronary artery disease in approximately 60% of patients. Meanwhile, up to two-thirds of these individuals have coronary microvascular dysfunction (CMD), which often explains their symptoms. CMD is a clinically important condition that affects women with chronic coronary syndrome much more often than men. In these patients, impaired myocardial flow reserve (MFR) is a reliable predictor of adverse clinical outcomes, even in the absence of visible perfusion defects. Positron emission tomography (PET) enables quantification of myocardial blood flow (MBF) and MFR, both of which play critical roles in the diagnosis of CMD, support accurate risk stratification, and may be useful for monitoring treatment response. Emerging radiotracers, such as ^18^F-flurpiridaz, offer favorable pharmacokinetics and practical logistical benefits, thereby improving clinical access to PET imaging. Parallel advances, including artificial intelligence-powered analytical tools and total-body PET, are expected to enhance reproducibility and efficiency, further integrating myocardial flow assessment into routine clinical workflows. As technology and evidence continue to evolve, PET-based MBF quantification is likely to become an essential tool in precision cardiovascular care, effectively closing the gap between physiology and clinical decision-making. This narrative review aims to provide an overview of coronary microvascular pathophysiology and the most up-to-date evidence regarding the use of PET-derived MBF quantification in the diagnosis and management of patients with CMD.

## 1. Introduction

Chest pain in patients without obstructive coronary artery disease (CAD), particularly in women, has long been recognized as a clinical challenge that continues to impose substantial economic, social, and healthcare costs [1,2]. In a large cohort study of symptomatic patients undergoing invasive coronary angiography (CAG) for suspected obstructive CAD, over half of them were found to have no obstructive CAD [3]. This patient population, defined as having ischemia with no obstructive coronary artery disease (INOCA) [4], experiences elevated morbidity and mortality, reduced quality of life [5], and a higher risk of major adverse cardiovascular event (MACE) incidence [6,7,8,9]. INOCA patients present with heterogeneous clinical features and multiple underlying causes. However, consistent evidence from multiple studies has identified coronary microvascular dysfunction (CMD) as the functional cause of angina in 50%–65% of these individuals [10,11,12]. At the mechanistic level, several pathogenetic mechanisms are thought to give rise to CMD, including impaired arteriolar vasodilation, inflammation driven by cardiovascular risk factors, vascular smooth muscle hypersensitivity, and thrombosis. These abnormalities collectively cause ischemia by impairing the normal increase in myocardial blood flow (MBF) that occurs when transitioning from rest to stress [13,14,15].

Epidemiologically, INOCA and CMD exhibit striking sex disparities, with women accounting for 60%–70% of all INOCA cases and experiencing a 2–3-fold higher prevalence of CMD compared to men, particularly after menopause [3]. The burden of CMD extends far beyond primary INOCA, as it impacts an estimated 50%–60% of patients with type 2 diabetes, 40%–50% of those with long-standing hypertension, and up to 80% of patients with hypertrophic cardiomyopathy [16,17]. Long-term prognostic data indicate that patients with confirmed CMD have a 1.5–2.5-fold increased risk of 5-year MACE incidence, including myocardial infarction, heart failure-related hospitalization, and cardiovascular death, with annual mortality rates approaching 3% in high-risk subgroups [7].

While single-photon emission computed tomography (SPECT) has long been the mainstay of myocardial perfusion imaging (MPI), it only enables the evaluation of relative changes in regional myocardial perfusion, whereas it is relatively inaccurate as a means of diagnosing global MBF impairment, particularly in patients with multivessel or microvascular disease [18,19]. Although the relatively recent introduction of dedicated cardiac cameras fitted with Cadmium-Zinc-Telluride (CZT) detectors has allowed for the calculation of absolute MBF and myocardial flow reserve (MFR) estimates using dynamic MPI [20,21], in recent decades, positron emission tomography (PET) has emerged as the gold-standard noninvasive method for simultaneously measuring absolute MBF (in mL/g/min) and the corresponding MFR (defined as stress MBF divided by rest MBF). PET also provides higher spatial resolution and reliable attenuation correction [22]. Importantly, the clinically meaningful prognostic value of MBF quantification has been consistently confirmed across diverse patient populations, both with and without obstructive CAD. This finding has helped reduce unnecessary CAG procedures in clinical surveillance protocols [23,24,25]. As PET technology becomes increasingly more widely used for MPI and MBF quantification, this has also significantly increased clinical awareness of microvascular angina.

This narrative review aims to provide a comprehensive overview of the technical aspects of PET-based MBF quantification and the pathophysiology of CMD, while also exploring the increasingly central role of PET imaging in the diagnosis and risk stratification of patients with CMD. We also highlight key knowledge gaps and outline future research directions in this rapidly evolving field.

## 2. Clinical Expression and Classification of Coronary Microvascular Dysfunction

Patients with CMD may experience typical exertional angina (retrosternal oppressive chest pain), atypical symptoms, or angina equivalents such as dyspnea on exertion or reduced exercise tolerance [26]. Microvascular angina (MVA), a clinical manifestation of myocardial ischemia in patients without flow-limiting CAD, often occurs during exercise but can also persist or predominate in the post-exercise recovery phase as the mismatch between myocardial oxygen supply and demand continues [27]. MVA may also present with atypical symptoms, such as prolonged retrosternal discomfort at rest or stabbing chest pain of variable duration. While both men and women experience similar signs and symptoms of MVA, it is more common in women, especially after menopause [28]. As nitrates primarily dilate large coronary arteries but have a fairly minimal impact on small arterioles, less marked symptomatic relief is generally achieved in patients with MVA as compared to those with obstructive CAD. Notably, in patients with myocardial bridging, nitrate therapy may paradoxically worsen systolic vessel compression and potentially increase anginal symptoms.

In 2007, Camici and Crea [29] proposed a clinical setting-based approach to CMD classification, defining four major subtypes of this condition. Type I CMD occurs in patients without obstructive CAD or myocardial disease, and is linked to conventional cardiovascular risk factors, including smoking, hypertension, diabetes, and dyslipidemia. Type 2 CMD is considered secondary to underlying myocardial diseases, including hypertrophic cardiomyopathy, dilated cardiomyopathy, aortic stenosis, and infiltrative heart disease. Type 3 CMD occurs in patients affected by obstructive CAD, including both those with stable CAD and those impacted by acute coronary syndromes. Notably, the ORBITA trial recently demonstrated that approximately 50% of patients continue to experience stable angina symptoms despite successful percutaneous coronary intervention (PCI) of hemodynamically significant epicardial lesions [30], and a substantial proportion of these individuals have underlying microvascular dysfunction [31]. Type 4 CMD cases are instances of iatrogenic CMD caused by distal embolization during coronary percutaneous revascularization procedures [29]. After successful revascularization, this subtype of CMD may present with delayed myocardial flow normalization that can persist for several weeks, with this delay being an independent predictor of MACE incidence [32]. Given that CMD can occur across a spectrum of clinical settings, ranging from minimal or absent atherosclerosis to both obstructive and non-obstructive CAD, Taqueti and Di Carli [33] proposed a simpler three-category classification framework acknowledging the overlapping clinical presentations and pathophysiological mechanisms of CMD across these different contexts, while also emphasizing the important prognostic interaction between CMD and CAD. The three categories proposed under this system are as follows: (1) CMD without CAD, (2) CMD with non-obstructive CAD, and (3) CMD with obstructive CAD [33]. In addition to highlighting the critical interaction between CMD severity and the extent of CAD, this system underscores their combined impact on MACE risk.

Uncertainties surrounding the definition of CMD prompted the Coronary Vasomotion Disorders International Study Group (COVADIS) to develop standardized diagnostic criteria for these disorders [34]. Based on COVADIS consensus recommendations compiled during their 2014 and 2015 summits, the diagnosis of CMD requires that individuals meet the following four criteria: (1) presence of symptoms suggestive of myocardial ischemia; (2) absence of obstructive CAD (<50% coronary diameter reduction and/or fractional flow reserve [FFR] >0.80); (3) objective evidence of myocardial ischemia; and (4) evidence of impaired coronary blood flow (CBF) reserve (Table 1). When all four criteria are fulfilled, a diagnosis of “definite MVA” is applied, whereas “suspected MVA” is diagnosed when criteria (1) and (2) are met, together with either criterion (3) or (4). Diagnostic testing that directly assesses coronary microvascular function is thus a cornerstone of CMD diagnosis, and detection of impaired hyperemic MBF or MFR has become an integral component of MVA diagnosis in patients presenting with chest pain. However, it is important to recognize the limitations of current classification systems. The Camici & Crea 4-type and Taqueti & Di Carli 3-type classifications were developed primarily based on clinical context and anatomical findings and do not take the quantitative physiological data afforded by PET into consideration, such that their utility for guiding targeted therapy based on underlying pathophysiology remains limited. Similarly, while the COVADIS criteria represent an important step toward standardization, they have several limitations. Most notably, the fourth criterion (“evidence of impaired coronary blood flow reserve”) does not specify the approach used to measure flow reserve, nor does it provide standardized cutoff values for different imaging modalities. This has contributed to significant variability in the approach to CMD diagnosis across studies, impeding efforts to compare results and pool data for meta-analyses. Additionally, the COVADIS criteria require objective evidence of myocardial ischemia, which can be challenging to obtain in patients with purely microvascular disease, given that diffuse, balanced ischemia can be difficult to detect using standard electrocardiographic and perfusion imaging techniques.

**Table 1.** **COVADIS clinical criteria for the diagnosis of MVA**.

**Symptoms suggestive of myocardial ischemia**
Effort and/or rest angina
Angina equivalents (i.e., shortness of breath)
**Absence of obstructive CAD (<50% coronary diameter reduction and/or fractional flow reserve [FFR] >0.80) by coronary computed tomography angiography or invasive coronary angiography**
**Objective evidence of myocardial ischemia**
Ischemic ECG changes during an episode of chest pain
Stress-induced chest pain and/or ischemic ECG changes with or without transient/reversible abnormal myocardial perfusion and/or wall motion abnormality
**Evidence of impaired coronary blood flow reserve**
Impaired coronary flow reserve (cut-off values from ≤2.0 to ≤2.5, depending on methodology)
Coronary microvascular spasm, defined as reproduction of symptoms and ischemic ECG shifts, but no epicardial spasm during acetylcholine testing
Abnormal coronary microvascular resistance indices (e.g., IMR >25)
Coronary slow-flow phenomenon, defined as TIMI frame count >25

Definitive MVA is only diagnosed if all 4 criteria are present. Suspected MVA is diagnosed if symptoms of ischemia with no obstructive coronary artery disease are present (criteria 1 and 2), but only objective evidence of myocardial ischemia (criteria 3) or evidence of impaired coronary blood flow reserve (criteria 4) alone. COVADIS, coronary vasomotion disorders international study group; CAD, coronary artery disease; MVA, microvascular angina; ECG, electrocardiogram; IMR, index of microcirculatory resistance; TIMI, thrombolysis in myocardial infarction.

The existing classification systems for CMD (Camici & Crea 4-type and Taqueti & Di Carli 3-type) were developed based on clinical context and anatomical findings, but do not incorporate the quantitative physiological data provided by PET. To address this gap, we propose establishing an integrated framework that reconciles these systems and links each CMD subtype to measurable PET signatures, prognostic risk, and preferred therapeutic approaches (Table 2). This framework addresses a critical limitation of current classifications by providing clinicians with a direct link between pathophysiology, imaging findings, and treatment decisions.

**Table 2.** **Integrated clinical-PET classification of CMD**.

Integrated CMD subtype	Core clinical features	PET quantitative signature	Prognostic risk	Preferred first-line therapy
Isolated functional CMD (no CAD)	Younger patients, anxiety, hypertension, tachycardia	Normal minimal microvascular resistance (MMR), elevated rest MBF (>0.8 mL/g/min), MFR 1.8–2.5	Low-intermediate	Beta-blockers, ivabradine, metabolic modulators
Isolated structural CMD (no CAD)	Older patients, long-standing hypertension, diabetes, HFpEF	Elevated MMR, normal rest MBF, stress MBF <1.8 mL/g/min, MFR <2.0	High	ACEI/ARBs, statins, SGLT2 inhibitors
CMD with non-obstructive CAD	Atherosclerotic risk factors, mild epicardial plaque	Mixed pattern: regional stress MBF defects with global MFR reduction	Intermediate-high	Intensive risk factor modification + anti-anginal therapy
CMD with obstructive CAD	Post-PCI persistent angina, multivessel disease	Reduced stress MBF in both stenotic and non-stenotic territories	Very high	Guideline-directed medical therapy + targeted microvascular interventions
Iatrogenic CMD	Recent PCI, periprocedural myocardial injury	Transient global reduction in stress MBF, gradual improvement over 4–6 weeks	Intermediate	Antiplatelet therapy, statins, short-term calcium channel blockers

CAD, coronary artery disease; CMD, coronary microvascular dysfunction; PET, positron emission tomography; MFR, myocardial flow reserve; MBF, myocardial blood flow; PCI, percutaneous coronary intervention; HFpEF, heart failure with preserved ejection fraction; ACEI, angiotensin-converting enzyme inhibitors; ARBs, angiotensin II receptor blockers; SGLT, sodium-glucose linked transporter.

This classification system is particularly valuable when differentiating between functional and structural CMD, which are characterized by distinct underlying mechanisms and require different treatment approaches. For example, patients with isolated functional CMD often have elevated resting MBF due to increased sympathetic tone, such that beta-blockers are the most appropriate first-line therapy. In contrast, patients with structural CMD have impaired vasodilatory reserve due to vascular remodeling and therefore attain greater benefit from therapies that improve endothelial function and vascular structure.

## 3. Pathophysiology of Coronary Microvascular Dysfunction

The coronary arteries are divided into two distinct anatomical compartments: epicardial segments and the coronary microcirculation (Fig. 1). The latter consists of pre-arterioles, arterioles, and capillaries [35]. Pre-arterioles range from 100 to 500 μm in diameter and primarily function to maintain perfusion pressure in downstream arterioles through endothelium-dependent vasodilatory mechanisms. An increase in pre-arteriolar CBF triggers vasodilation, whereas reduced flow causes vasoconstriction. Arterioles measure less than 100 μm in diameter and function by directly matching myocardial blood supply to myocardial oxygen demand. The collective actions of pre-arterioles and arterioles serve as the primary means of regulating coronary vascular resistance, which maintains stable CBF. They achieve this regulation by appropriately modulating the tone of vascular smooth muscle cells (VSMCs) in response to myocardial oxygen consumption. As the smallest component of the coronary microvasculature, capillaries measures ~10 μm in diameter and serve as the primary site for gas and nutrient exchange. As a disease entity, CMD encompasses all pathological processes affecting coronary blood vessels with diameters less than 500 μm, including vascular wall infiltration, extraluminal compression, sympathetic nerve dysfunction, and abnormal vascular remodeling. Despite extensive research, the precise mechanisms underlying these pathological changes remain incompletely understood [36]. Importantly, these deleterious processes are not mutually exclusive and often co-occur, resulting in a broad spectrum of functional and structural vascular abnormalities.

**Fig. 1.**
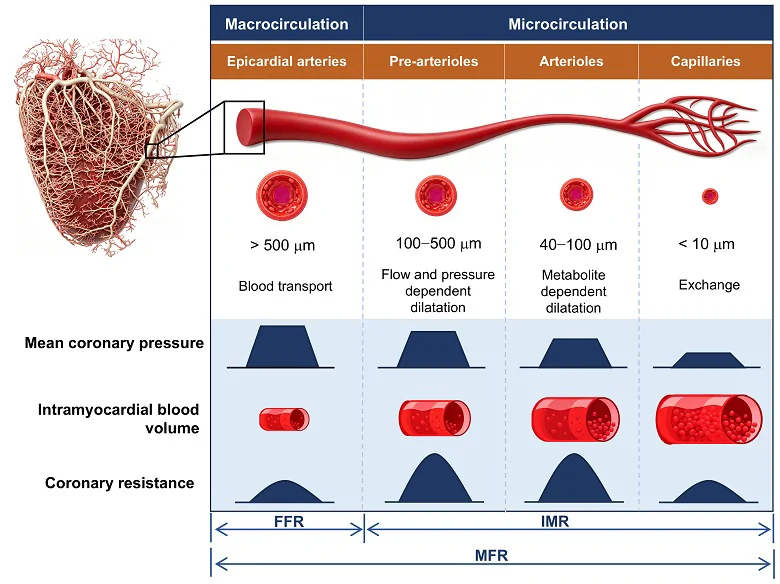
**Schematic overview of the coronary arterial system with associated functional and structural indices**. Under physiological conditions, arterioles and capillaries contain the largest fraction of IMBV, while pre-arterioles and arterioles serve as the primary determinants of coronary vascular resistance. Epicardial coronary arteries contribute negligibly to both total coronary resistance and IMBV. FFR, fractional flow reserve; IMR, index of microcirculatory resistance; MFR, myocardial flow reserve; IMBV, intramyocardial blood volume.

Endothelial dysfunction is a defining feature of CMD, marked by reduced nitric oxide (NO) bioavailability, increased oxidative stress, and the activation of endothelin-1 signaling and other vasoconstrictive pathways. This functional imbalance is widely regarded as the primary cause of impaired endothelium-dependent vasodilation and excessive vasoconstriction. Together, these abnormalities reduce MFR and cause myocardial ischemia, even when epicardial arteries appear normal on angiography [37].

Recent work has highlighted the heterogeneity that defines CMD, which consists of two distinct endotypes: structural CMD (primarily associated with abnormal hyperemic MBF) and functional CMD (primarily associated with abnormal resting MBF). Both endotypes are characterized by high rates of inducible ischemia and abnormal exercise responses, but differ in their underlying pathobiological mechanisms and can be clinically differentiated through the measurement of minimal microvascular resistance. Structural CMD is defined by elevated minimal microvascular resistance, which can result from microvascular structural changes such as capillary rarefaction or the impairment of endothelial NO synthase function. These processes, in turn, result in normal resting CBF but an impaired ability to sufficiently increase CBF in response to stress. In contrast, functional CMD is associated with normal minimal microvascular resistance, with affected patients exhibiting partially vasodilated vessels at rest, such that they are unable to increase their CBF further during stress [38]. Elevated resting CBF may arise due to increased myocardial oxygen demand and/or metabolic abnormalities, including arterial hypertension, tachycardia, metabolic syndrome, and obesity [39]. It remains unclear whether this abnormal resting state stems from a physiologically appropriate response to elevated myocardial oxygen demand at rest or is attributable to the abnormal autoregulation of neuronal NO synthase, which is the enzyme responsible for controlling basal CBF. From a therapeutic perspective, structural CMD may be most responsive to interventions that reduce afterload and improve vascular remodeling, whereas treatments for functional CMD should focus on optimizing basal myocardial metabolism. In clinical practice, PET-based MBF quantification provides a noninvasive approach to distinguishing between these two endotypes. Structural CMD is characterized by a disproportionate reduction in stress MBF relative to rest MBF, resulting in a low MFR with normal resting flow. In contrast, functional CMD typically presents with a normal or elevated resting MBF and a blunted stress response, leading to a reduced MFR despite relatively preserved absolute stress MBF values.

While this structural/functional endotype classification scheme provides valuable insight into the heterogeneity of CMD, it is important to acknowledge the limitations of this approach. Prognostic data supporting this classification strategy are primarily derived from the Inclusive Invasive Physiological Assessment in Angina Syndromes (ILIAS) registry [38], which was a retrospective, multicenter study with inherent selection bias. Definitely distinguishing between these two endotypes also necessitates the measurement of minimal microvascular resistance, which is not standardized across centers, and different calculation methods can yield significantly different results. Furthermore, while the ILIAS registry found that structural CMD was associated with a higher risk of MACE compared to functional CMD, this finding has not been independently validated in other large cohorts. Another important limitation is that many patients appear to have mixed CMD phenotypes with features of both structural and functional dysfunction, complicating classification efforts. Finally, inflammatory and thrombotic mechanisms have been estimated to account for <20% of the overall variability in microvascular function [40], underscoring significant gaps in our current understanding of CMD pathophysiology and suggesting that other, as of yet unidentified, mechanisms play a major role in the development of this condition.

Structural changes in the coronary microcirculation include pathological alterations such as arteriolar wall thickening, perivascular fibrosis, and microvascular rarefaction. These changes reduce the luminal cross-sectional area of vessels and increase minimal vascular resistance. These structural abnormalities are most commonly observed in patients with systemic hypertension, diabetes mellitus, and heart failure with preserved ejection fraction (HFpEF), as the reduced vessel compliance and fibrosis evident in these conditions impair vasodilatory reserve [41]. Physical narrowing of the intramyocardial arterioles can occur in response to extravascular compressive forces. Moreover, the tight coupling of myocardial oxygen consumption and CBF can be disrupted in response to impaired metabolic signaling, potentially leading to CMD in the context of left ventricular hypertrophy with elevated wall stress and interstitial edema in acute ischemic syndromes [42].

Inflammatory and thrombotic mechanisms also worsen microvascular dysfunction, much as observed in obstructive CAD. However, these mechanisms account for ≤20% of the variability in microvascular function, with most cases remaining unexplained [40]. In patients with acute coronary syndromes, distal microembolization and leukocyte infiltration cause microvascular obstruction. This increases infarct size and worsens clinical outcomes, even with successful epicardial reperfusion [43]. The inflammatory response also reduces NO bioavailability, forming a key mechanistic link between atherosclerosis and microvascular dysfunction. This leads to the impairment of both endothelium-dependent and endothelium-independent vasomotor function of the coronary microvasculature, thereby increasing the risk of myocardial ischemia.

Finally, neurohormonal dysregulation is emerging as an important modulator of microvascular tone. Sympathetic and parasympathetic nerves regulate microvascular tone by directly acting on VSMCs or by stimulating endothelial NO release [44]. The exact physiological role of vagal nerve control over CBF remains unclear. In experimental settings, intracoronary acetylcholine injection causes dilation of resistance vessels and a 3- to 4-fold increase in CBF, whereas this response is blunted or causes epicardial or microvascular coronary spasm in patients with coronary atherosclerosis [45]. Increased sympathetic activity, reduced parasympathetic input, and abnormal adrenergic receptor signaling can trigger dynamic microvascular vasoconstriction, which worsens existing structural and endothelial abnormalities [46,47].

## 4. PET Myocardial Blood Flow Quantification

### 4.1 PET Radiotracers

PET-based MBF quantification relies on dynamic imaging to track tracer kinetics from the arterial blood pool into myocardial tissue. This technique provides MBF measurements with a high degree of spatial and contrast resolution, lower radiation exposure, shorter scan times, and higher diagnostic accuracy compared to SPECT [48,49]. CZT-SPECT MBF quantification remains challenging due to nonlinear myocardial tracer uptake, although some attempts at K_1_ parameter estimation have been made [50].

Four commonly used PET tracers have been validated for MBF quantification: ^13^N-ammonia (^13^N-NH_3_), ^82^Rubidium (^82^Rb), ^15^O-water (^15^O-H_2_O), and the newly approved ^18^F-flurpiridaz (Table 3). The image quality and kinetic model assumptions associated with each of these tracers differ with tracer-specific half-life, extraction fraction, and production logistics. ^15^O-H_2_O is unique among these radiotracers in that it is inert and freely diffusible, such that it exhibits a linear relationship with MBF and near-complete myocardial extraction, establishing it as the physiological gold standard for MBF measurement against which all new radiotracers are validated. Despite these advantageous properties, its clinical use is largely restricted to research settings as it necessitates access to an on-site cyclotron, has an extremely short half-life (2.04 minutes), and exhibits high background blood pool activity. These factors preclude the generation of high-quality relative perfusion images [51]. While ^13^N-NH_3_ offers a high extraction fraction and excellent image quality, enabling comprehensive ischemia assessment, the need for on-site cyclotron access similarly limits its widespread clinical use. Notably, however, a compact ^13^N-NH_3_ production system has recently been developed and validated for clinical PET MPI in humans, offering a lower-cost alternative to conventional full-sized on-site cyclotrons [52]. Unlike ^15^O-H_2_O, which remains metabolically inert and freely diffusible, ^13^N-NH_3_ is subject to metabolic trapping.

**Table 3.** **Radiotracer characteristics, image acquisition for PET perfusion imaging, MBF quantification**.

	^82^Rubidium	^13^N-ammonia	^18^F-flurpiridaz	^15^O-water
Production	^82^Sr/^82^Rb generator	Onsite cyclotron	Regional cyclotron	Onsite cyclotron
Half-life	76 s	9.96 min	109.8 min	2.04 min
Extraction fraction^†^, %	≈60–65	≈80	≈94	≈100
Radiation exposure, mSv	~0.7	~1	~4.6	~0.4
Positron range, mm	8.6	2.53	1.03	4.14
Scan duration, min	6	10–20	10–20	5
Dose-2D, mCi	40–60	15–25	NA	40
Dose-3D, mCi	15–20	10–15	5	10
Interval between doses, min	10	30	NA	7
Image resolution	Lowest (~4–5 mm)	Intermediate-high (~3–4 mm)	Highest (~2–3 mm)	Intermediate (~3.5–4.5 mm)
Pharmacologic stress imaging protocol	Feasible	Feasible	Feasible	Feasible
Exercise imaging protocol	Not feasible	Feasible but not practical	Feasible	Not feasible
Stress MBF cutoff for ischemia, mL/g/min	1.5–1.8	1.8	1.8–2.0	2.3
MFR cutoff for ischemia	2.0	2.0	2.0	2.5
Uptake mechanism	Potassium analog, active transport via Na^+^/K^+^-ATPase	Passive diffusion + metabolic trapping by glutamine synthetase	Passive diffusion + mitochondrial metabolic trapping	Free diffusible tracer, no metabolic retention
Advantages	Not requiring a cyclotron on site Low radiation exposure	High myocardial extraction fraction Better spatial resolution than ^82^Rb Well-established clinical reference tracer	Flow-independent high extraction fraction (>90%) Long half-life Short positron range for superior image quality	High myocardial extraction fraction Accurate measurement of hyperemic MBF up to 5 mL/g/min Short scan time and low radiation dose
Limitations	Significant roll-off at high flows Low myocardial extraction fraction Long positron range limits spatial resolution	Requiring a cyclotron on site Short half-life Mild flow-dependent saturation at high MBF (>3 mL/g/min) Not compatible with exercise stress testing	Alterations in the metabolic state of the myocardium may affect its retention Limited long-term clinical data	Limited application to facilities with an on-site cyclotron Ultra-short half-life Poor spatial resolution

^†^Extraction fractions are listed for baseline MBF (≈1 mL/g/min). 2D, 2-dimensional; 3D, 3-dimensional; MBF, myocardial blood flow; MFR, myocardial flow reserve; NA, not applicable; PET, positron emission tomography.

^82^Rb has an extremely short half-life of 76 seconds, which allows for full rest-stress imaging and MBF quantification in less than 30 minutes. Additionally, in high-volume clinical centers, the ^82^Sr-^82^Rb generator can be used efficiently, making ^82^Rb a cost-effective choice for busy practices. Although ^82^Rb is widely available through generator systems, its reduced extraction fraction at high flow rates can lead to the underestimation of MBF if not properly corrected for [53]. Additionally, the spatial resolution of ^82^Rb is slightly lower than that of ^13^N-ammonia owing to its longer positron emission range [54].

^18^F-flurpiridaz is a novel PET radiotracer specifically designed to improve myocardial perfusion imaging [55]. ^18^F-flurpiridaz PET provides multiple clinical advantages compared with conventional SPECT imaging [56], including higher spatial resolution, faster imaging times, and the ability to accurately quantify MBF. These features allow clinicians to achieve greater diagnostic precision, facilitating improved patient care. Studies have shown that ^18^F-flurpiridaz PET affords greater sensitivity for the detection of coronary artery disease, especially in patient populations that have historically been difficult to image, including women, individuals with obesity, and those undergoing pharmacological stress testing. This improved sensitivity is particularly valuable in complex clinical situations such as CMD, where conventional imaging results are often inconclusive [57]. Finally, ^18^F-flurpiridaz exhibits a high first-pass extraction fraction of ~94% and a longer half-life of 109.8 minutes, allowing for its integration into exercise stress imaging protocols [58,59]. This longer half-life also allows for the production of ^18^F-flurpiridaz at regional cyclotron facilities followed by its distribution to remote imaging centers.

To be broadly effective, the myocardial uptake of an ideal quantitative MPI agent should be linearly proportional to MBF across a wide range of flow rates. Both ^15^O-H_2_O and ^18^F-flurpiridaz exhibit this favorable extraction profile. In contrast, the extraction fraction of ^82^Rb, and to a lesser degree ^13^N-NH_3_, decreases with increasing flow rates, although both can still be used to accurately measure MBF across a broad range of flow values by applying appropriate extraction fraction corrections to account for this “roll-off phenomenon” [60]. Additionally, an ideal tracer should have high first-pass extraction and minimal recirculation. For most clinically available radiotracers, two phenomena are observed with increasing coronary flow: (1) competitive back-diffusion of tracer into the bloodstream after initial myocardial uptake, or (2) saturation of the tracer uptake mechanism [61]. An ideal tracer should exhibit minimal roll-off in the context of high stress-induced flow rates. It should also be rapidly cleared from the blood pool and have kinetics that are not influenced by metabolic changes or the type of pharmacological stress agent used. Furthermore, an ideal tracer should exhibit low extracardiac uptake, particularly in the liver and lungs, given their cardiac proximity. Finally, an ideal perfusion agent should be safe without significant side effects, chemically stable, and cost-effective to manufacture.

While previous reviews have compared the technical characteristics of different PET radiotracers, they have not provided context-specific guidance for selecting the optimal tracer based on institutional resources and patient characteristics. To address this gap, we propose a practical decision tree for radiotracer selection (Table 4).

**Table 4.** **Evidence-based radiotracer selection decision tree**.

**1. For centers without on-site cyclotron access**
First-line: ^82^Rb for routine clinical cases, due to its low radiation exposure, short scan time, and generator-based availability
Preferred for obese patients or those requiring exercise stress: ^18^F-flurpiridaz, due to its high spatial resolution and long half-life
**2. For centers with on-site cyclotron access**
Clinical reference standard: ^13^N-NH_3_, due to its high extraction fraction and well-established clinical validation
Research applications or cases requiring extreme accuracy: ^15^O-water, due to its linear relationship with MBF across all flow rates
**3. Special patient populations**
Patients with breast attenuation: ^18^F-flurpiridaz or ^13^N-NH_3_, due to their superior spatial resolution compared to ^82^Rb
Patients requiring serial monitoring: ^82^Rb, due to its lowest radiation exposure
Patients with suspected balanced ischemia: ^15^O-water or ^18^F-flurpiridaz, due to their minimal roll-off at high flow rates

MBF, myocardial blood flow.

### 4.2 Flow Quantitation Methods

Prior to image acquisition, patients should rest quietly on the scanner bed for several minutes to prevent artificially elevated resting MBF. Baseline heart rate and blood pressure should also be documented at this time. Three key steps are critical to ensure accurate data capture, especially for the arterial input function. First, the PET scanner should be started in advance for system preparation before tracer administration, and rest PET acquisition commences after tracer injection. Second, data acquisition should be continued until reaching myocardial tracer equilibrium. Third, a standardized time interval between pharmacologic stressor administration and tracer injection should be maintained. MBF quantification requires dynamic list-mode acquisition to create time-activity curves (TACs) for both the blood pool (arterial input function) and myocardium, followed by kinetic modeling to calculate MBF (mL/g/min) and MFR. Two primary approaches to modeling are used: compartmental modeling, which matches TACs to tracer-specific kinetic parameters (e.g., K_1_, K_2_), and simplified retention modeling, which relates late-phase myocardial uptake to integrated blood-pool activity. Simplified retention models are based on the assumption that tracer washout and metabolism are negligible during early imaging phases, obviating the need to calculate K_2_ and K_3_. This simplification eliminates the complex curve-fitting process required to calculate K_1_ in compartmental modeling, making this approach better suited to routine clinical use. PET-based calculation of absolute MBF is achieved with the formula K_1_ = MBF × extraction fraction**. **However, varying degrees of nonlinearity have been observed between K_1_ and MBF at high flow rates for different tracers [62]. Freely diffusible tracers such as ^15^O-H_2_O can be accurately modeled using a single compartment model because they quickly reach equilibrium between intra- and extravascular spaces, and their washout constant (K_2_) directly reflects MBF (Fig. 2).

**Fig. 2.**
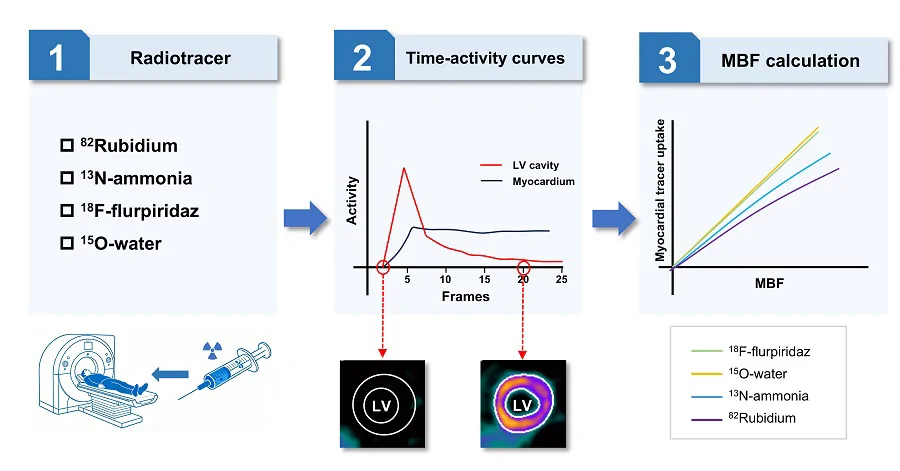
**Schematic overview of the measurement of MBF using PET**. The association between myocardial tracer uptake and MBF for PET radiotracers demonstrates a roll-off phenomenon at high flow rates, as the uptake of some radiotracers becomes diffusion-limited at higher flows. This contributes to reduced accuracy when quantifying hyperemic coronary flow and MFR. PET, positron emission tomography; MBF, myocardial blood flow; LV, left ventricular.

Several corrections are required during tracer kinetic modeling using 1- to 3-compartment models and operational equations to generate absolute MBF values [63,64]. These include corrections for physical decay of positron-emitting tracers, partial volume effects (which cause underestimation of true myocardial tissue concentrations and are typically adjusted for by assuming a uniform myocardial wall thickness of 1 cm), and radioactivity spillover between the left ventricular blood pool and myocardium. This approach for quantifying MBF with multiple positron-emitting flow tracers has been validated against gold-standard microsphere measurements. Additionally, from the final frame or static image of the PET scan (e.g., after 600 seconds), the relative distribution of myocardial radiotracer uptake and perfusion can be evaluated visually or semiquantitatively, other than when using ^15^O-H_2_O, which relies solely on quantitative blood flow measurements [63]. While visual or semi-quantitative analyses, such as summed stress and rest scores [65], remain the standard of care, they frequently fail to detect balanced or diffuse ischemia [62]. Quantitative assessment, in contrast, allows for the more comprehensive evaluation of myocardial perfusion. The presence of stress-induced regional perfusion deficits accompanied by concordant reductions in the context of absolute hyperemic MBF and MFR values is thus typically indicative of hemodynamically significant obstructive CAD lesions. In contrast, homogeneous radiotracer uptake following stress and preserved absolute hyperemic MBF and MFR values effectively preclude the presence of flow-limiting epicardial lesions and are associated with excellent clinical outcomes [66]. Normal stress‑rest myocardial perfusion images with homogenous radiotracer uptake and normal wall motion, together with reduced hyperemic MBF and/or MFR, are highly suggestive of CMD. A standardized workflow for dynamic PET MBF quantification is presented in Fig. 3.

**Fig. 3.**
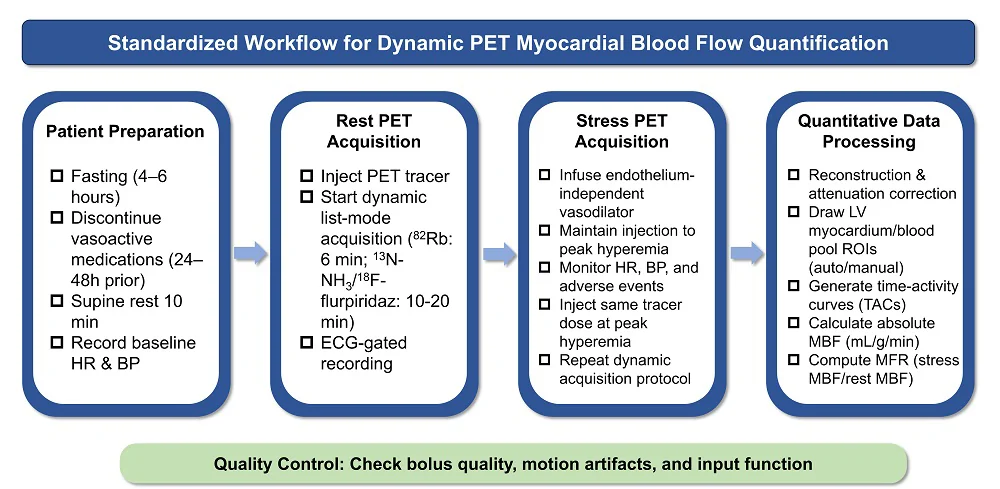
**Standardized workflow for dynamic PET-based absolute myocardial blood flow quantification in clinical practice and research**. PET, positron emission tomography; MBF, myocardial blood flow; HR, heart rate; BP, blood pressure; ECG, electrocardiogram; ROI, region of interest; TAC, time-activity curve; MFR, myocardial flow reserve.

 However, there are important limitations to current quantification methods. While accurate, compartmental modeling is both computationally intensive and noise-sensitive, whereas the assumption of negligible tracer washout implicit in simplified retention models may not be valid in the context of high flow rates or abnormal myocardial metabolism [62]. Partial volume effect correction, which typically assumes a uniform 1 cm wall thickness, is particularly problematic in patients with hypertrophy or thinning. MBF measurements have also been reported to vary significantly across software platforms, even when using identical raw data [67]. The accuracy of these methods is also critically dependent on high-quality arterial input function generation, which is susceptible to bolus variability, motion artifacts, and partial volume effects in the left ventricular cavity.

When utilizing commercially available software packages, quantitative MBF assessment can be seamlessly integrated into standard PET perfusion imaging protocols without the need for any increases in scan time or radiation exposure [67]. Stress MBF is best measured during near-maximal hyperemia using endothelial-independent vasodilators (adenosine, dipyridamole, or regadenoson), which are favored over sympathetic agonists (dobutamine) or exercise stress testing as they produce a more consistent and standardized hyperemic response [68]. Exercise myocardial perfusion PET is rarely used in clinical practice, as the short half-lives of most currently approved radiotracers do not afford sufficient time between tracer injection during peak treadmill exercise and patient transfer/positioning for PET imaging. Supine bicycle exercise performed directly in the PET scanner is possible, but can give rise to substantial motion artifacts. When performing serial assessments, the use of the same stress agent allows for the longitudinal comparison of MBF values. MBF quantification can also be impacted by several technical factors, including ROI positioning and bolus quality, which must be carefully evaluated for each individual study (Fig. 4).

**Fig. 4.**
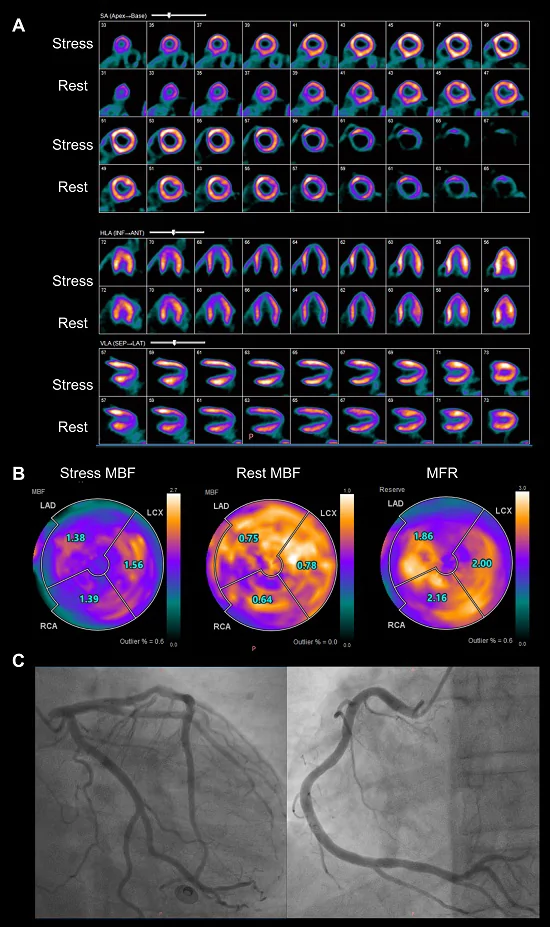
**Cardiac PET imaging and invasive coronary angiography in a 59-year-old woman with coronary microvascular dysfunction**. (A) Hyperemic flow stimulation with regadenoson and rest ^13^N-ammonia PET images in corresponding short-axis (top), horizontal long-axis (middle), and vertical long-axis (bottom) slices. (B) Quantification of myocardial blood flow. Regional MBF quantification of hyperemic MBF and at rest with resulting MFR calculation using ^13^N-ammonia PET and tracer kinetic modeling. A global reduction in hyperemic MBF was observed across all three coronary territories (all <1.85 mL/g/min). Hyperemic MBF was less impaired in the LCx territory, such that MFR was considered borderline-normal (2.00). Preserved MFR was evident in the RCA territory (2.16) despite a significant reduction in hyperemic MBF, with this finding being explained by the relatively lower resting MBF for this territory (0.64 mL/g/min). (C) Invasive coronary angiography confirmed the absence of clinically significant coronary artery stenoses. MFR, myocardial flow reserve; PET, positron emission tomography; MBF, myocardial blood flow; LAD, left anterior descending; LCx, left circumflex; RCA, right coronary artery.

## 5. PET-Based Diagnosis of Coronary Microvascular Dysfunction

Current guidelines from the American College of Cardiology (ACC) and American Heart Association (AHA) [69] recommend the use of non-invasive approaches to CMD assessment in lieu of invasive strategies. Accordingly, only a few centers in the United States routinely perform invasive evaluation of microvascular dysfunction, with most CMD diagnoses relying on noninvasive testing using PET MPI and MBF quantification.

Clinical criteria for the diagnosis of MVA were recently published by COVADIS. These criteria have since been integrated into clinical frameworks to streamline diagnosis, registry development, and standardized recruitment for clinical trials [70]. Four core criteria were proposed, with PET imaging being suitable for the noninvasive fulfillment of the fourth criterion (Table 1). A common diagnostic challenge for PET MPI studies arises when patients exhibit normal perfusion imaging accompanied by global reductions in both MFR and stress MBF. Three main factors may account for this finding: (1) suboptimal response to pharmacological stress, (2) severe diffuse epicardial CAD, and (3) microvascular dysfunction [71]. Effectively differentiating among these three potential causes necessitates the careful interpretation of quantitative flow data in the context of clinical and imaging findings. To address this common clinical challenge and provide actionable guidance for practicing clinicians, we have developed a structured interpretation decision tree that outlines specific, evidence-based criteria for distinguishing between these three entities (Table 5). Although microvascular disease most often presents in the form of diffusely reduced stress MBF and MFR, regional stress MBF and MFR reductions accompanied by perfusion defects can also occur. Microvascular disease may also be the basis for a subset of false-positive results on MPI or other forms of non-invasive ischemia testing. Indeed, in a significant proportion of tests deemed false-positive due to the absence of obstructive disease (e.g., FFR >0.8), microvascular dysfunction can be confirmed invasively using the index of microcirculatory resistance (IMR), CFR, or the resistance reserve ratio (RRR) [31].

**Table 5.** **Structured interpretation decision tree for global MFR/MBF reduction with normal perfusion images**.

Step	Clinical/Imaging criteria	Diagnosis	Recommended next steps
Step 1: Rule out clinically significant hypotension (e.g., SBP <90 mmHg, symptomatic drop >20 mmHg) as abnormal markers	• Heart rate increase <10 bpm from baseline • Systolic blood pressure decrease >10 mmHg during stress • Patient history of severe asthma/COPD • Recent caffeine or theophylline ingestion (<12 hours) • Incomplete regadenoson injection • Absence of splenic switch-off sign on stress images	Suboptimal pharmacological stress	• Repeat stress test with alternative agent (adenosine/dipyridamole) • Ensure proper patient preparation before repeat testing
Step 2: Rule out severe diffuse epicardial CAD	• High cardiovascular risk profile (diabetes, long-standing hypertension, smoking) • Coronary artery calcium score >400 Agatston units • CT angiography showing diffuse coronary calcification (≥3 vessels) • MFR <1.5 with global stress MBF <1.5 mL/g/min • Mild, subtle regional perfusion heterogeneity	Severe diffuse epicardial CAD	• Refer for invasive coronary angiography with FFR measurement • Intensive guideline-directed medical therapy
Step 3: Diagnose CMD	• All criteria from Step 1 and Step 2 are negative • Typical or atypical angina symptoms • Female sex (postmenopausal) • MFR 1.5–2.5 with global stress MBF <1.8 mL/g/min • Homogeneous perfusion without regional defects	CMD	• Initiate targeted anti-anginal therapy (beta-blockers, ACEI/ARBs) • Intensive risk factor modification • 6-month follow-up PET to assess treatment response

The <1.8 mL/g/min threshold is a general cutoff for initial CMD screening. For definitive diagnosis, tracer‑specific stress MBF cutoffs presented in Table 3 should be applied. CAD, coronary artery disease; CMD, coronary microvascular dysfunction; PET, positron emission tomography; MFR, myocardial flow reserve; MBF, myocardial blood flow; ACEI, angiotensin-converting enzyme inhibitors; ARBs, angiotensin II receptor blockers; COPD, chronic obstructive pulmonary disease; FFR, fractional flow reserve.

The presence of reduced hyperemic MBF and MFR is nonspecific, as it can result from flow-limiting epicardial lesions, CMD, or both [72]. The appropriate clinical context must thus be considered when interpreting the presence of regional reductions in hyperemic MBF and/or MFR, taking myocardial perfusion images, coronary anatomy data, and individual cardiovascular risk profiles into account.

In addition to the structured decision tree outlined in Table 5, several key technical and interpretive considerations are essential to guide the accurate assessment of quantitative PET data. The first step in ensuring appropriate quality control is the careful inspection of TACs generated during dynamic acquisition. A valid left ventricular blood pool input function requires a sharp, well-defined peak within 30–60 seconds of tracer injection followed by rapid exponential decline, while a broad, delayed, or low-amplitude peak is indicative of poor bolus quality that may cause MBF to be significantly underestimated. Suboptimal pharmacological stress remains the most common cause of inaccurately detected reductions in myocardial flow reserve values, and while hemodynamic criteria provide useful guidance, the splenic switch-off sign is now recognized as a more objective imaging marker of adequate vasodilator response. Specifically, the administration of regadenoson or adenosine normally induces splenic vasoconstriction, resulting in markedly reduced splenic radiotracer uptake on stress images compared to rest, with the absence of this finding being strongly suggestive of inadequate stress even in patients with apparently normal hemodynamic changes.

Integrating functional PET data with coronary computed tomography angiography (CCTA)-derived anatomical information can further improve diagnostic accuracy. In patients with globally reduced flow reserve and normal perfusion images, extensive coronary calcification (calcium score >400 Agatston units) or diffuse non-obstructive plaque on CCTA is associated with a greater likelihood of underlying epicardial disease, whereas normal coronary anatomy on CCTA strongly supports a diagnosis of coronary microvascular dysfunction. This combined approach allows for the comprehensive evaluation of both anatomical and functional coronary status, allowing for more informed clinical decision-making.

Individuals at highest risk for developing CMD include women and those with conditions such as hypertension, diabetes mellitus (DM), left ventricular hypertrophy, slow coronary flow on angiography, infiltrative heart diseases, obesity, insulin resistance, and long COVID [73,74,75,76]. Given the high and increasing prevalence of hypertension, Xu et al. [77] showed that impaired MFR is associated with cardiovascular risk factors, elevated cardiac biomarkers, abnormal cardiac structure, and adverse clinical events in patients with hypertension but no obstructive CAD. Patel et al. [16] further reported that impaired MFR is common and associated with a greater risk of mortality among 2730 patients with DM who are free of known CAD [16]. Studies have also reported an association between poor glycemic control in type 2 DM and a higher prevalence of CMD characterized by reduced CFR, while IMR and RRR levels do not differ significantly between groups [78].

PET has also been extensively validated against other invasive and noninvasive tests used to assess ischemic burden in patients with CAD or non-ischemic cardiomyopathies [79,80,81]. In their study of 23 patients with chronic CAD using ^13^N‑NH_3_ PET‑MPI with quantitative MBF analysis and CAG with FFR measurement, Imami et al. [82] found that hyperemic MBF in normal-perfusion regions can accurately assess CMD severity. In a head-to-head comparison with ^15^O-H_2_O PET, Agostini et al. [83] evaluated the feasibility of MBF and MFR measurement using dynamic CZT-SPECT and determined that CZT-SPECT overestimates MBF. MBF and MFR measurements thus enable differentiation between microvascular and macrovascular disease, as confirmed by multiple studies [84,85,86,87]. Additionally, patchy, heterogeneous resting myocardial perfusion quantified using Markovian homogeneity analysis allows for the prediction of mild stress-induced perfusion abnormalities. These findings are consistent with CMD, which may serve as an early marker of preclinical CAD suitable for preventive interventions [88].

In addition to obstructive CAD and microvascular dysfunction, several other factors can influence hyperemic MBF and MFR values, including technical determinants and patient-related variables. Technical determinants such as PET scanner technology, radiotracer selection, and choice of kinetic modeling approach can impact PET flow quantification. Patient-related variables such as age, sex, and hemodynamic status can similarly impact MBF. For example, a gradual decline in hyperemic MBF and an increase in resting MBF occur in older adults, leading to a nonpathological reduction in MFR [89]. Complex interactions between focal stenosis, diffuse epicardial coronary narrowing, altered endothelial shear stress, and microvascular dysfunction often complicate definitive diagnosis and management in women relative to men when using standard noninvasive or invasive anatomical and physiological tests. When interpreted in light of clinical context, however, quantitative MBF and MFR can generally distinguish between the complex interacting pathophysiologies of microvascular, diffuse epicardial, and focal stenotic CAD allowing clinicians to guide aggressive risk factor modification and targeted interventions for large regions of severe stress-induced hypoperfusion identified by quantitative PET [90].

Notably, reductions in MFR may stem from increased resting MBF and/or decreased hyperemic MBF. For example, elevated resting flow caused by hypertension or anxiety can lead to reduced MFR, even in the absence of significant CAD. Indeed, Shan et al. [91] recently identified anxiety as a risk factor for CMD during mental stress in patients following PCI. Furthermore, both the magnitude and rate of MBF reduction were positively correlated with anxiety severity in patients with INOCA [92]. Arterial hypertension, increased myocardial contractility, tachycardia, or elevated left ventricular wall stress all increase myocardial oxygen demand and consequently resting MBF. Conversely, three main causes may contribute to reductions in hyperemic MBF: flow-limiting downstream effects of focal epicardial lesions, microvascular disease associated with cardiovascular risk factors, and increased extravascular compressive forces caused by elevated left ventricular pressure in patients with hypertension or congestive heart failure. This complicates efforts to define a single optimal threshold for diagnosing microvascular dysfunction across all patient populations. Nonetheless, current guidelines state that MFR values below 2.0–2.5 are indicative of microvascular dysfunction [70]. These thresholds, which are widely used in clinical practice, were derived from healthy control populations by calculating the mean MFR and subtracting two standard deviations.

Despite their widespread use, the implementation of rigid one-size-fits-all cutoffs is inherently limiting. MFR reduction may reflect elevated resting MBF due to hypertension, tachycardia, or anxiety rather than true microvascular dysfunction [91,92]. Furthermore, physiological declines in MBF and MFR are evident with age and differ by sex, despite the absence of any routinely employed age- or sex-specific thresholds [89]. Most validation studies comparing PET to invasive IMR have been small, single-center investigations [82,83]. While PET can reliably detect global CMD, its ability to identify regional microvascular abnormalities remains less well established, underscoring the need for caution when interpreting regional flow reductions to avoid misclassification of diffuse epicardial disease or suboptimal stress response as CMD [71].

More recently, a landmark prospective multicenter study of 1001 patients with angina with non-obstructive coronary arteries (ANOCA) across nine centers in Europe and North America provided new insight into disease heterogeneity while additionally proposing a comprehensive pathophysiological classification scheme based on invasive testing of coronary vasomotor function. In this study, the authors used a standardized protocol combining adenosine and intracoronary acetylcholine testing to measure CFR and index of microvascular resistance (IMR), leading to the identification of eight distinct endotypes of ANOCA. Three of these endotypes were defined by abnormal adenosine responses: high resting CBF, high microvascular resistance, and compensated high resistance. The remaining five endotypes were identified by abnormal acetylcholine responses: epicardial coronary spasm, microvascular spasm, endothelial dysfunction, ischemia without measurable hemodynamic changes, and enhanced cardiac nociception. Importantly, this protocol detected at least one abnormality in 77% of patients, with 12% exhibiting multiple overlapping endotypes, underscoring the complex and heterogeneous nature of ANOCA [93].

### Prognostic Value of PET

PET-based MBF and MFR assessment provides important prognostic information in patients with CMD. Multivariate regression models have identified an independent relationship between PET-derived MFR and cardiovascular events [85], outperforming body mass index (BMI) and traditional risk factors, including left ventricular ejection fraction and summed stress score, in risk discrimination. In a large cohort of 4029 patients referred for rest/stress PET myocardial perfusion imaging, MFR also predicted cardiovascular mortality more effectively than did maximal MBF [94]. This association remained significant even when adjusting for traditional cardiovascular risk factors, left ventricular ejection fraction, myocardial scar and ischemia, rate-pressure product, radiotracer and stress agent type, and post-scan revascularization.

Murthy et al. [95] showed that the addition of MFR analysis to standard risk assessment led to the correct reclassification of 35% of patients with intermediate risk scores. A model integrating PET-derived MFR along with biomarkers including high-density lipoprotein (HDL), red blood cell distribution width (RDW), glycated hemoglobin/hemoglobin A1c (HbA1c), and B-type natriuretic peptide (BNP) reportedly provides a robust means of confirming CMD diagnosis [84]. In contrast, renin-angiotensin-aldosterone system (RAAS) activity measured based on plasma renin concentration and endothelin-1 levels is not associated with CMD and should not be used for diagnostic purposes [86]. Another PET-derived index, impaired modified coronary flow capacity (mCFC), is independently associated with both cardiovascular death and MACE in patients with suspected CAD and without obstructive disease (hazard ratio, 10.7; 95% confidence interval, 1.0–106.0; *p* = 0.04 and HR, 9.5; 95% confidence interval, 2.5–36.2; *p* < 0.001, respectively) [96]. Taqueti et al. [97] also found that women with reduced MFR on PET and no obstructive CAD exhibit a significantly elevated adjusted risk of cardiovascular events. This same research group also determined that abnormal flow reserve in patients without CAD is associated with diastolic dysfunction and incident heart failure [94].

Based on the evidence outlined above, recent American and European guidelines recommend PET MPI with MBF quantification for patients with an intermediate to high pretest likelihood of CAD [58,98]. This strategy is conducive to the detection of microvascular CAD, which is particularly important in symptomatic women and patients with diabetes. PET/CT achieves 98% accuracy when differentiating between CMD and epicardial stenoses, while exposing patients to a radiation dose of under 10 mSv [99]. This limited radiation exposure makes it a safe option for routine clinical use. These benefits are further enhanced by technological advances such as new 3D PET scanners that have further reduced radiation doses while maintaining high quantitative accuracy [100].

The prognostic value of PET flow quantification has also been confirmed in patients with conditions and risk factors linked to a high prevalence of microvascular disease [39,101]. Cardiovascular risk factors and other yet unidentified factors may initially cause endothelial injury and functional impairment [102,103,104]. Over time, these changes also impact the function of vascular smooth muscle, leading to CMD, which in turn promotes structural CAD onset. Herzog et al. [102], for instance, demonstrated the incremental prognostic value of adding MFR to stress ^13^N-ammonia PET perfusion imaging. In patients with normal stress-rest PET perfusion images, an MFR ≥2.0 conferred a 3-year event-free survival. Notably, even in patients with stress-induced regional perfusion defects, an MFR <2.0 provided incremental prognostic information beyond that afforded by standard perfusion imaging when predicting adverse cardiovascular events. Ziadi et al. [105] also obtained similar findings through their larger study of 704 patients with and without obstructive CAD who underwent ^82^Rb-PET for myocardial perfusion and MBF assessment. In patients with diabetes, coronary microvascular dysfunction is associated with an annual mortality rate of 7.6%, which is significantly higher than of non-diabetic patients with established CAD (1.3% per year) [106]. In this population, MFR has been shown to improve risk stratification beyond conventional clinical assessment, left ventricular ejection fraction, and MPI-derived measures of ischemia and scar burden. Similarly, impaired flow reserve is highly prevalent and associated with increased all-cause mortality among patients with chronic kidney disease [107] and those with dialysis-dependent end-stage renal disease [108]. PET has also been used as a means of evaluating CMD in valvular heart diseases, most notably aortic stenosis [109]. MFR and stress MBF are correlated with markers of myocardial injury and wall stress, indicating that MFR may offer utility as a sensitive early marker of myocardial decompensation in these patients [110]. Finally, the incremental prognostic value of reduced MFR for cardiac death has been demonstrated in several high-risk populations, including women [97], as well as patients with cardiometabolic disease [111], hypertrophic obstructive cardiomyopathy [17], cardiac allograft vasculopathy [112], and ischemic or idiopathic cardiomyopathy [113]. Giacobbe et al. [114] further extended these findings through a systematic review in which they characterized the pathophysiological and prognostic roles of CMD across the full spectrum of non-ischemic cardiomyopathies, including dilated cardiomyopathy, Takotsubo syndrome, arrhythmogenic right ventricular cardiomyopathy, and infiltrative disorders such as cardiac amyloidosis and Anderson-Fabry disease. They determined that CMD often precedes overt structural abnormalities in these conditions and that PET-derived MBF quantification provides unique value for early detection and risk stratification, particularly in cases of infiltrative cardiomyopathy in which microvascular dysfunction may be the earliest detectable sign of cardiac involvement.

Overall, the available evidence demonstrates that PET-derived impairments in hyperemic MBF and/or MFR provide robust prognostic information across diverse patient populations. This emphasizes CMD as an integrated index of the total vascular stress burden imposed by cardiovascular risk factors. Despite consistent prognostic associations across studies, however, several limitations must be acknowledged. Nearly all available evidence is observational, precluding causal inferences between impaired MBF/MFR and adverse outcomes. Significant heterogeneity exists in study design, patient populations, and MFR cutoff values (ranging from 1.5 to 2.5), limiting meta-analytic comparability. Most studies have not evaluated the incremental prognostic value of MFR over contemporary biomarkers such as high-sensitivity troponin or NT-proBNP. Additionally, data on patient-centered outcomes (symptoms, quality of life) remain limited, and while interventions can improve MBF/MFR, there is no definitive evidence that these improvements translate to any corresponding reduction in clinical events [115,116,117].

PET offers a unique opportunity to monitor CMD responses to both behavioral interventions, such as exercise, diet, and weight loss, as well as medical therapies aimed at restoring microvascular function, improving symptoms, and potentially enhancing clinical outcomes [118]. A large body of evidence suggests that risk factor modification and medical therapy can improve or even normalize hyperemic MBF and MFR in asymptomatic individuals [81]. Interventions with demonstrated efficacy include statins, insulin-sensitizing thiazolidinediones, optimized glycemic control in diabetes, angiotensin-converting enzyme inhibitors, angiotensin II type 1 receptor blockers, hormone replacement therapy in postmenopausal women, regular exercise, and gastric bypass-induced weight loss. However, definitive evidence for causal relationships between these interventions, PET-measured improvements in MBF, and subsequent changes in symptoms or clinical outcomes remains limited. For patients with microvascular angina, the CorMicA trial demonstrated that invasive CMD assessment-guided targeted medical therapy resulted in significant improvements in angina relief and quality of life at 6 months, with no increase in adverse events [119].

The integration of cardiac PET into the diagnostic pathway for selected patient populations thus enhances clinical decision-making by providing detailed insights into both epicardial and microvascular coronary function (Fig. 5). This is particularly crucial for those patients who remain symptomatic despite the absence of any significant epicardial coronary stenosis on conventional imaging as it allows clinicians to implement personalized medical interventions with greater odds of improving patient outcomes. Cardiac PET is thus more than a tool for the diagnosis of ischemia, serving as an important modality for assessing coronary microvascular health, making it highly valuable for the management of complex cardiac cases. Cardiac PET should be considered when evaluating patients with stable chest pain, multiple comorbidities, and no obstructive CAD to ensure a comprehensive evaluation and guide effective management strategies. This approach reflects current guidelines and best practices, aiming to provide optimize care for patients whose cardiac symptoms may be related to microvascular dysfunction with optimal care.

**Fig. 5.**
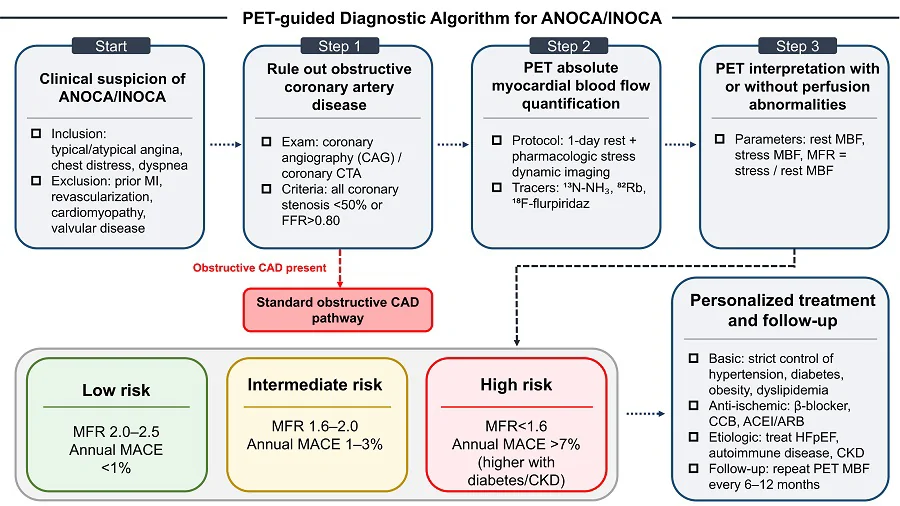
**PET-guided diagnostic and prognostic algorithm for patients with suspected angina and ischemia with no obstructive coronary artery disease**. ANOCA, angina with no obstructive coronary artery disease; INOCA, ischemia with no obstructive coronary artery disease; CAD, coronary artery disease; CAG, invasive coronary angiography; CTA, computed tomography angiography; FFR, fractional flow reserve; MFR, myocardial flow reserve; MACE, major adverse cardiovascular events; HFpEF, heart failure with preserved ejection fraction; CKD, chronic kidney disease; ACEI, angiotensin-converting enzyme inhibitor; ARB, angiotensin II receptor blocker; CCB, calcium channel blocker.

## 6. Future Directions

Given its distinct pathophysiology and prognostic profile, CMD should be differentiated from other causes of chest pain in patients without obstructive CAD, including coronary vasospasm and non-cardiac etiologies. PET-derived quantitative MBF and MFR have revolutionized noninvasive cardiac imaging by providing detailed physiological insights into microvascular disease that far exceed those obtained from relative perfusion imaging alone. However, the prioritization of six key areas will be vital to fully realize the clinical potential of this technology.

### 6.1 Standardize Acquisition Protocols and Reporting Practices

A high degree of heterogeneity in research methodology and outcome measures is evident among the studies included in this review. Modest variations in PET MBF values can arise depending on the radiotracer, scanner model, stress agent, and analysis software used. In the absence of standardized protocols, the comparability of multicenter studies remains limited. The use of MFR can partially mitigate the variations in absolute blood flow estimates arising from differences in imaging modality, stress agent, and quantification method. However, residual differences persist, influencing the relationship between MFR and clinical endpoints, including mortality [120]. This variability is a likely cause of inconsistencies in research outcomes across studies and contributes to the lack of any consensus diagnostic definitions or cut-off values for CMD across noninvasive imaging modalities. The American Society of Nuclear Cardiology (ASNC) has launched multiple guidelines and educational initiatives with the goal of addressing this issue. Standardizing acquisition and analytical methodologies and integrating MBF/MFR into routine clinical reports will contribute to greater clinician confidence and the more widespread adoption of quantitative flow assessment in daily practice. There remains an urgent need for large multicenter validation studies, and the standardization of acquisition and analytical protocols is essential to minimize heterogeneity and improve comparability among approaches, given that current modalities are not yet interchangeable [48,62,121,122].

### 6.2 Generate Outcome-Driven Clinical Evidence

As emphasized throughout this review, the prognostic value of PET-derived MFR is supported by evidence from many observational studies. Randomized controlled trials to determine whether flow-guided management strategies improve clinical outcomes, thus represent a critical next step for the field. These trials will help accelerate clinical adoption and ultimately improve cardiovascular outcomes, particularly in high-risk populations, including women, patients with diabetes, and those with HFpEF. Recent studies have demonstrated that cardiomyocyte injury and myocardial stiffness caused by CMD may contribute to the pathophysiology of HFpEF [118]. A growing body of evidence suggests that microvascular endothelial dysfunction, reduced NO bioavailability, and enhanced cytokine signaling may contribute to the increased microvascular inflammation and myocardial fibrosis observed in HFpEF [123]. Clinically, reduced CFR is associated with both diastolic dysfunction and a five-fold increased risk of HFpEF hospitalization. Clinical studies also show that HFpEF is more prevalent in women than in men [124]. The association between CMD and HFpEF remains an important area for future research, particularly with respect to the evaluation of sex-based differences. To optimize preventative and therapeutic strategies, a more comprehensive understanding of CMD risk factors is required. While traditional comorbid risk factors, including hypertension, hyperlipidemia, and diabetes, are well established, the roles of autoimmune conditions and adverse pregnancy outcomes are far less understood. Furthermore, additional research is needed to evaluate novel risk markers such as high-sensitivity C-reactive protein, lipoproteins, interleukins, and other inflammatory mediators.

### 6.3 Integrate Artificial Intelligence (AI) into PET MBF Quantification

AI-based quantification tools are helping improve the speed, reproducibility, and accessibility of flow imaging, particularly through automated MBF and MFR calculation. Tasks that previously required manual expert intervention, including myocardial segmentation, motion correction, time-activity curve extraction, and kinetic modeling, can now be automated using AI-powered software platforms. These systems have been shown to produce reproducible results comparable to expert analysis while significantly reducing processing time [125]. Convolutional neural networks (CNNs) trained on large PET datasets enable fully automated quantitative analysis, which expands access to flow imaging beyond specialized academic centers [126]. AI is also enabling novel diagnostic and predictive applications. Machine learning (ML) models have been shown to detect subtle perfusion abnormalities, identify microvascular dysfunction, and even predict PET-derived MFR from standard 12-lead ECGs, offering a promising opportunity for risk stratification in resource-limited settings where advanced imaging is unavailable. For instance, recent ML models have been trained to predict PET-derived MFR directly from routine 12-lead ECGs, demonstrating robust prognostic performance for MACE [127]. Additionally, AI-driven motion correction and reconstruction methods improve image quality, which is particularly valuable in challenging cases such as arrhythmias or impaired breath-holding capacity. These techniques have also been shown to improve the diagnostic accuracy of tracers such as ^18^F-flurpiridaz. Importantly, AI also has the potential to facilitate standardization across clinical centers. Historically, variability in kinetic models and analysis software has limited reproducibility between centers. AI-based systems incorporating built-in quality control features and integrated with structured reporting tools, electronic health records, and multicenter registries can help standardize image interpretation, accelerate research, and facilitate widespread implementation of quantitative PET imaging in clinical care [58,62]. Widespread adoption of AI-assisted workflows, including automated preprocessing, arterial input function (AIF) detection and modeling [128], MBF map generation [129], and prognostication [130], is expected to further simplify the analysis pipeline and reduce operator dependence. These technologies must be validated through prospective clinical trials and registry studies to confirm their clinical utility and minimize practice variation.

### 6.4 Develop Effective Disease-Modifying Therapies

At present, there are no evidence-based disease-modifying therapies for CMD. To address this gap in clinical care, future studies are needed to examine the effects of therapies on symptoms, quality of life, and clinical outcomes, with a focus on both established anti-anginal agents used in microvascular angina and novel therapies targeting the specific pathophysiology of CMD. No rigorous evaluation of common anti-anginal therapies, such as beta-blockers, calcium channel blockers, nitrates, and statins, has been conducted to date in patients with CMD. Some research suggests that intensive treatment with statins, angiotensin-converting enzyme inhibitors (ACEIs), or angiotensin II receptor blockers (ARBs) may be beneficial. Notably, several studies have also demonstrated improvements in PET-derived MBF following a range of interventions, including the initiation of lipid-lowering therapy [115], antihyperglycemic therapy in type 2 diabetes [116], antihypertensive treatment [117], and bariatric surgery [131]. While most of these studies enrolled asymptomatic subjects who did not have microvascular angina by definition, their results nonetheless suggest that PET flow quantification offers a means of monitoring therapeutic response in microvascular disease. Given its noninvasive nature and good reproducibility, PET-derived MFR represents a promising tool for evaluating therapeutic efficacy in patients with CMD and enabling personalized precision care. However, this MFR-guided management approach and the therapies themselves remain to be investigated at length in large-scale randomized clinical trials. Encouragingly, several ongoing studies are currently investigating the prevalence, detection, and treatment of CMD.

### 6.5 Contextualize MBF Interpretation

MBF measurements are influenced by patient-specific factors, including age, sex, blood pressure, anemia, and metabolic risk factors. Rigid, one-size-fits-all cutoffs can be misleading. By establishing large, population-stratified reference databases, it will be possible to achieve personalized, risk-adjusted interpretation conducive to greater diagnostic and prognostic precision.

### 6.6 Expand PET Access and Infrastructure

Despite its clinical value, there are notable practical barriers to the widespread implementation of PET MBF quantification, including relatively high cost, inconsistent reimbursement policies, restricted radiotracer supply, and limited availability outside academic tertiary centers. Limited radiotracer availability and the need for on-site generators or cyclotrons are the primary factors limiting its availability to academic tertiary settings. The development of ^18^F-flurpiridaz may help overcome many of these barriers, as its 110-minute half-life is amenable to unit-dose distribution and incorporation into routine exercise stress protocols, lowering barriers to broader PET adoption. Policy initiatives, provider education, and reimbursement support focused on disseminating best practices, demonstrating clinical value, and supporting the establishment of new cardiac PET services will help further expand access.

## 7. Conclusion

CMD impacts an estimated two-thirds of patients with nonobstructive CAD, resulting in recurrent angina symptoms that continue to impose a major healthcare burden. Despite its relevance to patient care, this condition is frequently underrecognized by clinicians. For affected individuals, PET-based MBF quantification has emerged as a major advance in noninvasive cardiac imaging, offering the potential to significantly improve clinical outcomes and quality of life for this unique and often underserved population. As the field progresses toward widespread clinical adoption, there is a need to shift focus away from demonstrations of feasibility in favor of prioritizing scalable, standardized, and outcome-driven clinical implementation. AI-assisted analytical workflows will play an important role in accelerating the broader clinical adoption of quantitative MPI. Ultimately, integrating quantitative MBF assessment into routine clinical care will enable the field to move beyond anatomical evaluation to physiological assessment, providing a robust diagnostic tool for evaluating microvascular function and monitoring therapeutic response.

## Abbreviations

CMD, coronary microvascular dysfunction; MFR, myocardial flow reserve; PET, positron emission tomography; MBF, myocardial blood flow; CAD, coronary artery disease; CAG, coronary angiography; INOCA, ischemia with no obstructive coronary artery disease; MACE, major adverse cardiovascular events.
